# Damping Control and Experiment on Active Hydro-Pneumatic Suspension of Sprayer Based on Genetic Algorithm Optimization

**DOI:** 10.3389/fnbot.2021.707390

**Published:** 2021-07-13

**Authors:** Chaofan Qiao, Haojun Wen, Xinyue Liu, Guangyan Wang

**Affiliations:** ^1^School of Mechanical and Electrical Engineering, Shihezi University, Shihezi, China; ^2^Key Laboratory of Northwest Agricultural Equipment, Ministry of Agriculture and Rural Affairs, Shihezi, China

**Keywords:** high ground clearance self-propelled sprayer, active hydro-pneumatic suspension, genetic algorithm, fuzzy PID, experiment on hydro-pneumatic suspension of sprayer control

## Abstract

High ground clearance self-propelled sprayers usually work in complex road conditions. Due to the large body mass, wide spray boom breath and high center of gravity, the body and spray boom swing sharply during work, which affects operation quality and even endangers safety. This paper proposes a control plan for timely-started active hydro-pneumatic suspension, and designs a fuzzy PID control system based on genetic algorithm optimization. First, MATLAB software is used to simulate and analyze the model, so that the fuzzy PID control optimized by genetic algorithm is obtained. When the sprayer drives on D-grade road, as the speed increases, in comparison between the damping effect of the active suspension and traditional passive suspension, the corresponding root mean square value of vehicle body vibration acceleration decreases by 11.36 and 12.36%, respectively. On the E-grade road surface, with the increase of speed, the corresponding root mean square value of vehicle body vibration acceleration decreases by 13.25 and 14.89%, respectively. Based on indoor bench experiments, the proposed control strategy was verified. Under field road excitation, when the sprayer traveled at 5 km/h, the root mean square acceleration values of the passive and active suspensions were 1.080 and 0.847 m/s^2^, respectively; when the sprayer traveled at 8 km/h, the root mean square acceleration values of the passive and active suspensions were 1.412 and 1.125 m/s^2^, respectively, with the root mean square values of vibration acceleration reduced by 21.57 and 20.33%, respectively. Under sand-gravel road condition, when the sprayer traveled at 5 km/h, the root mean square acceleration values of the passive and active suspensions were 1.149 and 0.891 m/s^2^, respectively; when the sprayer traveled at 8 km/h, the root-mean-square acceleration values of the passive and active suspensions were 1.572 and 1.229 m/s^2^, respectively, with the root mean square values of vibration acceleration reduced by 22.45 and 21.82%, respectively. During the active control process, the suspension displacement is always kept within the limited range, and as the vehicle speed and road surface level increase, the active suspension has a significantly better damping effect than the passive suspension, which proves effectiveness of the active damping scheme.

## Introduction

As an important intelligent plant protection machine (Sharda et al., 2013), high ground clearance self-propelled sprayer has wide applications, high efficiency, and precise plant protection operations, have a broad vision of application. Sprayers usually work on bumpy field roads. The environment is complicated. The operation involves huge amplitude. The high center of mass and ground clearance of the whole vehicle make it prone to severe vibration and affect the operation safety (Ferreira et al., 2010; Li et al., 2018). The vehicle may turn sideways in severe cases^1^^,^^2^. When working, the chassis suspension damping can reduce the spray rod vibration and improve the spray quality; when driving on the road, the suspension system can improve the ride comfort and operational stability of the vehicle (Sun et al., 2013; Chen et al., 2019). However, there are few studies on the suspension system of high ground clearance self-propelled sprayer. Therefore, it is of great significance to design an active suspension with excellent damping performance.

At present, most researches on sprayer suspension systems focus on passive and semi-active types mainly with spiral springs, air springs, and oil-air springs. Compared with air springs and spiral springs, hydro-pneumatic springs feature less space occupation, simple structure, convenient installation, and control adjustment (Melzi et al., 2014). AGCO equips its Challenger RG600 series sprayer with a passive hydro-pneumatic suspension system^3^ (Zatrieb and Kasler, 2012), which cushions the impact of uneven ground and reduces the vibration of the spray boom when the sprayer passes through uneven ground. Regarding semi-active hydro-pneumatic suspension that relies on spring damping adjustment to acquire the ideal damping performance, research is still focused on the suspension parameters, vehicle matching, adjustable damping scheme design, and damping control strategy (Poussot-Vassal, 2012; Tseng and Hrovat, 2015). The controllable high-pressure oil source is introduced into the pressure chamber of the hydro-pneumatic spring to charge and discharge oil to the hydraulic cylinder at an appropriate time, thereby changing its instantaneous pressure. Such active hydro-pneumatic spring is integrated into suspension to form an active hydro-pneumatic suspension. By injecting energy according to system requirements, active suspension simultaneously changes the instantaneous stiffness and damping of the spring to acquire the optimal damping effect (Wang, 2020; Wu et al., 2020). Compared with the semi-active suspension, the active suspension increases the energy supply source. If turned on at the right time, it has relatively small energy consumption and simpler overall structure, which significantly improves the overall performance of the sprayer.

The active hydro-pneumatic suspension system consists of the suspension mechanical structure and the active force implementation part. The hydro-pneumatic suspension system has obvious parameter uncertainty and relatively strong non-linear characteristics (Kim and Lee, 2011; Saglam and Unlusoy, 2016; Halfinann and Hung, 2017; Halfmann et al., 2017). The traditional method is to use a linear controller to act on the suspension system, which has low control accuracy and limited system performance effect, thus unable to meet the requirements (Wang and Suh, 2017), Use genetic algorithm to optimize target parameters and improve system performance (Li et al., 2005; Du and Wei, 2011). In the control process, the sensitivity of the ride comfort index and the attitude stability index to different weighting coefficients is analyzed for the active suspension vibration damping performance. With vehicle ride comfort as the index, the system parameters are optimized and estimated to set the controller's fault-tolerant control (Han et al., 2017), robust control (Tan, 2016), adaptive control (Senthil Kumar et al., 2018), sliding mode control (Guan et al., 2016) methods, thereby understanding control of the hydro-pneumatic suspension.

To solve the problem of severe vibration of high ground clearance self-propelled sprayer under complex environmental conditions, especially under large ground undulations, this paper takes the 1/4 active hydro-pneumatic suspension model as the research object, and proposes a timely-started active hydro-pneumatic suspension scheme to design a fuzzy PID control system based on genetic algorithm optimization. The control system applies fuzzy PID control method to deal with non-linearity in hydro-pneumatic spring charging and discharging. The genetic algorithm is used to optimize the fuzzy rules so that the fuzzy controller more reasonably controls the PID parameters. Through modeling and simulation of the active hydro-pneumatic suspension system, the control effect after the genetic algorithm optimization is analyzed, and the active hydro-pneumatic suspension experiment table is used to perform control test on the active hydro-pneumatic suspension, which verifies the designed damping scheme.

## Active Hydro-Pneumatic Suspension and Dynamic Model

**Figure 1:**
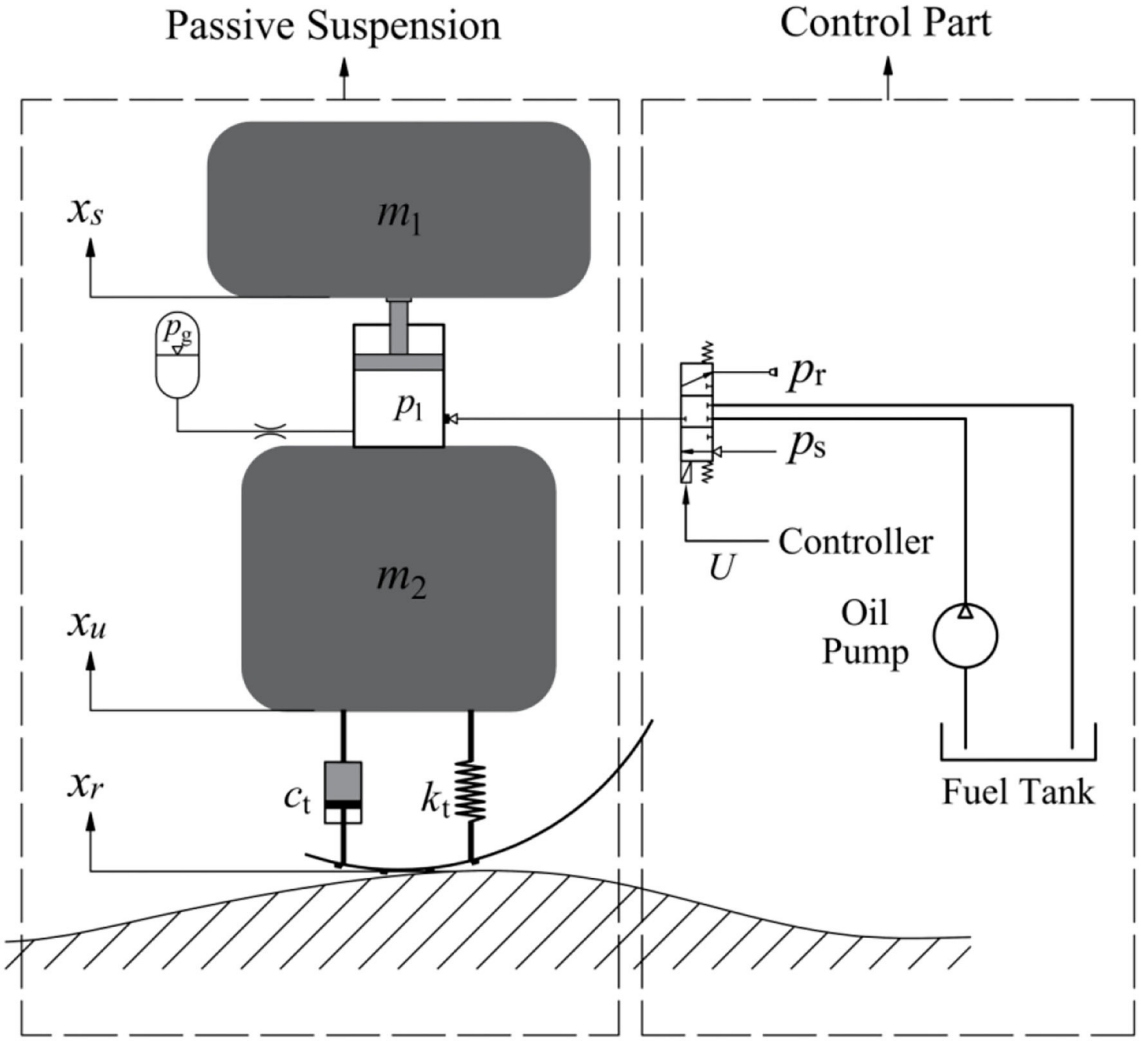
Dynamic model of active hydro-pneumatic suspension.

The structure of the active oil-pneumatic suspension system of the self-propelled sprayer is shown in Figure 1. The system is composed of two parts. The dashed line on the left shows the passive hydro-pneumatic suspension, and the dashed line on the right shows the suspension control part. By controlling the proportional solenoid valve, the hydro-pneumatic spring charges and discharges oil in real time to adjust the hydro-pneumatic suspension and achieve damping of the active suspension. In the figure, *m*_1_ and *m*_2_ are respectively 1/4 sprung mass and unsprung mass; *x*_*s*_, *x*_*u*_, *x*_*r*_ are respectively the sprung mass displacement, unsprung mass displacement and suspension input displacement of the road surface unevenness during the movement; *c*_*t*_ is the tire equivalent damping coefficient; *k*_*t*_ is the tire equivalent stiffness coefficient; *P*_1_ is the pressure state of the oil chamber, *P*_*s*_ is the fuel supply pressure, *P*_*r*_ is the outlet pressure when the fuel is discharged, and U is the control electrical signal.

It can be seen from Figure 1 that when the sprayer drives on a flat road, the solenoid valve is in the neutral position, cutting off the exchange between the spring and the fuel tank oil. At this time, the entire suspension system of the sprayer is equivalent to a passive suspension. When the sprayer travels on bumpy road, the solenoid valve is controlled to open, and the Fuzzy-PID control solenoid valve optimized by genetic algorithm directly acts on the cylinder of the hydro-pneumatic spring of the actuating mechanism to charge and discharge the oil in real time, so that hydro-pneumatic spring force is controllable, thus achieving damping of active suspension. Due to the targeted active control of opening and closure, the suspension reduces energy consumption as much as possible while ensuring the operation quality and smooth driving of the sprayer.

According to the thin-walled orifice theory in fluid mechanics, if fluid flows through a fixed orifice under a certain pressure, a certain pressure difference will be generated. *Q*_*g*_ is the flow through the throttle valve between the oil cylinder and the gas tank, that is, the flow through the orifice. According to the thin-walled orifice flow formula, the flow rate *Q*_*g*_ can be calculated as

(1) $$\begin{array}{l} Q_{g}=\{\begin{array}{c} C_{d}A_{k}\cdot {\left(\frac{2\cdot \left(P_{g}-P_{1}\right)}{\rho }\right)}^{1/2}\;P_{g}\geq P_{1}\;\\ {-C}_{d}A_{k}\cdot {\left(\frac{2\cdot \left(P_{1}-P_{g}\right)}{\rho }\right)}^{1/2}\;P_{g}<P_{1}\; \end{array} \end{array}$$

Where, *P*_1_ represents the pressure state of the oil chamber, *P*_*g*_ represents the pressure of the gas in the hydro-pneumatic spring accumulator; *C*_*d*_ is the flow coefficient, ρ is the oil density, and *A*_*k*_ is the effective flow area of the throttle valve hole. When *P*_*g*_ ≥ *P*_1_, it means that the oil flows into the cylinder; when *P*_*g*_ < *P*_1_, it means that the oil flows out of the cylinder.

By adjusting the proportional solenoid valve, the active and passive working modes of the hydro-pneumatic suspension can be switched, so that the charging and discharging process of the hydro-pneumatic spring and the oil flow *Q*_ν_ can be controlled to achieve the purpose of controlling the pressure of the hydro-pneumatic spring. *Q*_ν_ is the oil flow input and output from the external oil source to the hydro-pneumatic spring, and formula of the flow *Q*_ν_ is determined as

(2) $$\begin{array}{l} Q_{\nu }=\{\begin{array}{cc} C_{d}A_{c}{\left(\frac{2\left(P_{s}-P_{1}\right)}{\rho }\right)}^{\frac{1}{2}} & P_{s}\geq P_{1}\;\\ C_{d}A_{c}{\left(\frac{2\left(P_{1}-P_{r}\right)}{\rho }\right)}^{\frac{1}{2}} & P_{1}\geq P_{r}\; \end{array} \end{array}$$

Where, *P*_*s*_ is the oil supply pressure, *P*_*r*_ is the outlet pressure when the oil is discharged; *A*_*c*_ is the valve port area of the proportional solenoid valve, which has a certain relationship with the voltage input to the solenoid valve. When *P*_*s*_ ≥ *P*_1_, it means the hydro-pneumatic spring filling process; when *P*_1_ ≥ *P*_*r*_, it means the hydro-pneumatic spring unloading process.

The pressure change Ṗ_1_ in the hydraulic cylinder is

(3) $$\begin{array}{l} {\dot{P}}_{1}=\frac{\beta_{e}}{V_{10}+A_{1}\left(x_{s}-x_{u}\right)}\left(Q_{g}+Q_{\nu }-A_{1}\left({\dot{x}}_{s}-{\dot{x}}_{u}\right)\right) \end{array}$$

Where, β_*e*_ is the equivalent bulk elastic modulus of hydraulic oil, *V*_10_ is the initial oil storage volume of the oil cylinder, and *A*_1_ is the area of the oil chamber.

In the working process of the hydro-pneumatic spring, compressibility of the hydraulic oil can be ignored compared to the compressibility of the gas, so that the gas pressure *P*_*g*_ in the hydro-pneumatic spring accumulator can be calculated as

(4) $$\begin{array}{l} P_{g}=\frac{P_{g0}{V_{go}}^{n}}{{\left(V_{g0}+\int Q_{g}dt\right)}^{n}} \end{array}$$

Where, n is the gas adiabatic index, whose value depends on the conditions of the heat exchange process between the gas and the outside world. Under intense vibration, the gas in the spring cannot timely exchange heat with the outside world, which can be regarded as adiabatic change process in analysis. At this time, n normally takes a value of about 1.4. Under slow vibration, the gas has enough time to exchange heat with the outside world, which can be considered as an isothermal change process. At this time, the value of n is generally 1; *V*_*go*_, *P*_*go*_ are the initial values of volume state and absolute pressure in the hydro-pneumatic spring accumulator.

### Analysis on the Stiffness Characteristics of the Hydro-Pneumatic Spring

The working chamber of the hydro-pneumatic spring cylinder usually uses nitrogen as the filling gas, and its characteristics are very close to the ideal gas. Therefore, gas compression and expansion inside the cylinder can be calculated according to the law of ideal gas state change. It is assumed that the oil is not compressible. Then, the stiffness load *F*_*k*_ of the hydro-pneumatic spring is

(5) $$\begin{array}{l} F_{k}=\frac{A_{1}P_{go}{V_{go}}^{n}}{{\left(V_{g0}-A_{1}x\right)}^{n}} \end{array}$$

Where, *x* represents the relative displacement between the piston rod and the cylinder. It is stipulated that *x* is positive under compression and negative under tension.

By deriving the displacement of Equation (5), the stiffness coefficient K of the hydro-pneumatic spring can be calculated as

(6) $$\begin{array}{l} \text{K}=\frac{dF_{k}}{dx}=\frac{n{A_{1}}^{2}P_{go}{V_{go}}^{n}}{{\left(V_{g0}-A_{1}x\right)}^{n+1}} \end{array}$$

Hence, it can be known that the overall stiffness of the hydro-pneumatic spring presents relatively prominent non-linear characteristic with the change of the relative displacement between the piston rod and the cylinder barrel, and the stiffness force under compression is significantly greater than that under tension, which exhibits asymmetric characteristics.

### Analysis on the Damping Characteristics of the Hydro-Pneumatic Spring

Under the dynamic excitation of the hydro-pneumatic spring, due to the pressure difference between the hydraulic cylinder and the accumulator oil chamber, the oil flows back and forth through the damping hole, generates heat and then dissipates, thus consuming the energy of vibration and resulting in damping characteristics of the hydro-pneumatic spring. The damping effect of the hydro-pneumatic spring has two main sources: the first part is the damping caused by the hydro-pneumatic spring damping hole, which is the primary damping; the second part is the friction between the piston and the hydraulic cylinder. Due to the oil lubrication effect, it is much smaller compared to damping force of the damping hole and does not play a major role in damping, so it can be ignored in the analysis. Then, the damping force of the hydro-pneumatic spring is

(7) $$\begin{array}{l} F_{c}=\frac{{P_{1}C}_{d}A_{k}\cdot sgn\left(P_{g0}-P_{1}\right)\cdot {\left(\frac{2\cdot |P_{g0}-P_{1}|}{\rho }\right)}^{1/2}}{\dot{x}}-mg \end{array}$$

Where, *m* is the total sprung mass of the piston and its attached objects, *ẋ* represents the operating speed of the piston rod, *ẋ* < 0 indicates that the hydro-pneumatic spring is stretched, *ẋ* > 0 indicates that the hydro-pneumatic spring is compressed. The symbolic function *sgn*(*x*) in Equation (7) (x) is defined as:

(8) $$\begin{array}{l} sgn\left(x\right)=\{\begin{array}{cc} 1 & x\geq 0\\ -1 & x<0 \end{array} \end{array}$$

By derivation of the speed in Equation (7), we can get the damping coefficient C of the hydro-pneumatic spring:

(9) $$\begin{array}{l} C=\frac{\text{d}{\text{F}}_{\text{c}}}{d\dot{x}}=-\frac{{P_{1}C}_{d}A_{k}\cdot sgn\left(P_{g0}-P_{1}\right)\cdot {\left(\frac{2\cdot |P_{g0}-P_{1}|}{\rho }\right)}^{1/2}}{{\dot{x}}^{2}} \end{array}$$

Hence, it can be known that damping of the hydro-pneumatic spring exhibits obvious non-linear characteristics with the change in operating speed of the piston rod, and asymmetric characteristics are exhibited. That is, the damping coefficient in the compression stroke is obviously smaller than that in the extension stroke.

By synthesizing Newton's law of motion, the system motion differential equation of two-degree-of-freedom model of the 1/4 vehicle active hydro-pneumatic suspension can be obtained as:

(10) $$\begin{array}{l} \{\begin{array}{c} m_{1}{\ddot{x}}_{s}-F+m_{1}g=0\\ m_{2}{\ddot{x}}_{u}+F+k_{t}\left(x_{u}-x_{r}\right)+c_{t}\left({\dot{x}}_{u}-{\dot{x}}_{r}\right)-m_{2}g=0 \end{array} \end{array}$$

The force *F* of the hydraulic cylinder in Equation (10) is

(11) $$\begin{array}{l} F=A_{1}nP_{g0}{V_{g0}}^{-n}\int Q_{\nu }dt-nP_{g0}{V_{g0}}^{-n}{A_{1}}^{2}\left(x_{s}-x_{u}\right)\\ \;-C_{se}{A_{1}}^{2}\left({\dot{x}}_{S}-{\dot{x}}_{u}\right)+A_{1}C_{se}Q_{\nu } \end{array}$$

Where, *C*_*se*_ is the flow pressure coefficient of the flow restriction orifice.

From the above mathematical model, it can be known that the charging and discharging process of the hydro-pneumatic spring is discontinuous, and the parameters of the entire hydro-pneumatic spring control system vary with time, showing strong non-linearity. Therefore, non-linear control methods should be adopted.

Based on the state space, the mathematical model of the mechanical part of the active hydro-pneumatic suspension is converted into the state space form. The state variables are selected as

(12) $$\begin{array}{l} x_{1}=x_{s}-x_{u}\\ x_{2}=x_{u}-x_{r}\\ x_{3}={\dot{x}}_{s}\\ x_{4}={\dot{x}}_{u}\\ \;x={\left[x_{1}\;x_{2}\;x_{3}\;x_{4}\right]}^{T} \end{array}$$

Define the disturbance input as the speed *w*(*t*) = *ẋ*_*r*_(*t*) caused by road unevenness. The control input is *u*(*t*) = *U*, and the control output is

(13) $$\begin{array}{l} Z_{1}\left(t\right)={\ddot{x}}_{s}\\ Z_{2}\left(t\right)=\left(x_{s}-x_{u}\right)/x_{\max}\\ Z_{3}\left(t\right)=\left(k_{t}x_{u}-k_{t}x_{r}\right)/\left(m_{1}g+m_{2}g\right)\\ \;y\left(t\right)={\left[x_{s}-x_{u}\;{\ddot{x}}_{s}\right]}^{T} \end{array}$$

The system state equation is converted to:

(14) $$\begin{array}{l} \dot{x}\left(t\right)=Ax\left(t\right)+Bw\left(t\right)+B_{1}u\left(t\right)\\ Z_{1}\left(t\right)=Cx\left(t\right)+Dw\left(t\right)+D_{1}u\left(t\right)\\ Z_{2}\left(t\right)=C_{1}x\left(t\right)\\ Z_{3}\left(t\right)=C_{2}x\left(t\right)\\ \;y\left(t\right)=C_{3}x\left(t\right)+D_{2}w\left(t\right)+D_{3}u\left(t\right) \end{array}$$

Where

$$\begin{array}{l} \;A=\left[\begin{array}{cc} \begin{array}{cc} 0 & 0\\ 0 & 0 \end{array} & \begin{array}{cc} 1 & -1\\ 0 & 1 \end{array}\\ \begin{array}{cc} 0 & 0\\ 0 & -\frac{k_{t}}{m_{2}} \end{array} & \begin{array}{cc} 0 & 0\\ 0 & -\frac{c_{t}}{m_{2}} \end{array} \end{array}\right]\\ \;B={\left[\begin{array}{cccc} 0 & -1 & 0 & \frac{c_{t}}{m_{2}} \end{array}\right]}^{T}\\ B_{1}={\left[\begin{array}{cccc} 0 & 0 & \frac{A_{1}}{m_{1}} & -\frac{A_{1}}{m_{2}} \end{array}\right]}^{T}\\ \;C=\left[\begin{array}{cccc} 0 & 0 & 0 & 0 \end{array}\right]\\ \;D=0\\ D_{1}=\frac{A_{1}}{m_{1}}\\ C_{1}=\left[\begin{array}{cccc} \frac{1}{x_{\max}} & 0 & 0 & 0 \end{array}\right]\\ C_{2}=\left[\begin{array}{cccc} 0 & \frac{k_{t}}{\left(m_{1}+m_{2}\right)g} & 0 & 0 \end{array}\right]\\ C_{3}=\left[\begin{array}{cccc} 1 & 0 & 0 & 0\\ 0 & 0 & 0 & 0 \end{array}\right]\\ D_{2}={\left[\begin{array}{cc} 0 & 0 \end{array}\right]}^{T}\\ D_{3}={\left[\begin{array}{cc} 0 & \frac{A_{1}}{m_{1}} \end{array}\right]}^{T} \end{array}$$

Where

*Z*_1_(*t*)—represents the vertical acceleration of the vehicle body

*Z*_2_(*t*)—represents the travel limit of the suspension

*Z*_3_(*t*)—represents the ratio between dynamic and static load

0—zero matrix

*x*_max_—Maximum stroke of hydro-pneumatic spring

*y*(*t*)—suspension displacement and suspension acceleration

*Z*_1_(*t*) represents the vertical acceleration of the vehicle body. For the main controlled output, the control purpose is to make the output as small as possible to achieve the purpose of stabilizing the vehicle body vibration and actively reducing vibration. *Z*_2_(*t*) represents the suspension stroke limit. To avoid damage to the suspension parts during the control process, the expansion and contraction of the hydro-pneumatic spring should be smaller than the designed maximum stroke, that is, *Z*_2_(*t*) < 1. *Z*_3_(*t*) represents the tire dynamic and static load ratio. To ensure reliable tire adhesion during the control process, there must always be positive pressure between the tire and the ground during driving, that is, *Z*_3_(*t*) < 1 should be met.

## Control Principle

**Figure 2:**
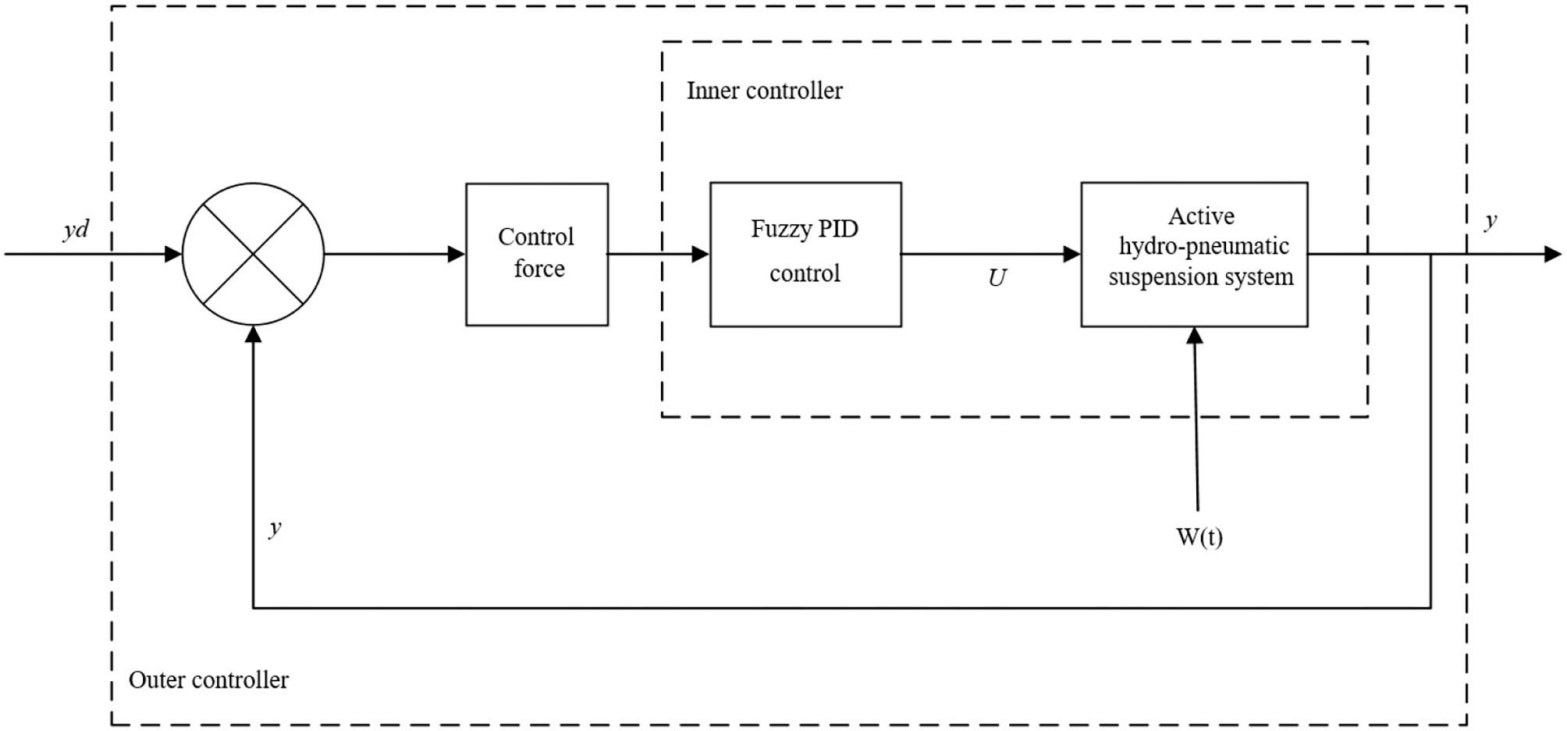
Active hydro-pneumatic suspension control scheme.

The outer controller is designed to calculate the expected control force required by the suspension system based on several states of the system, and the inner controller converts the expected control force output by the outer controller into the control voltage required by the proportional solenoid valve. Using Fuzzy-PID control method, the proportional solenoid valve is adjusted in time to control the in and out flow of the hydraulic oil of the hydro-pneumatic spring, thus achieving the desired control force output by the outer controller, as shown in Figure 2. In the figure, the vector *y* is the state output of the controlled system, and the vector *yd* is the expected output.

### Fuzzy PID Controller Design

Fuzzy PID control features good robustness, small overshoot and capability to solve non-linear factors, so it is also an effective way to solve non-linear complex systems. Accordingly, this paper uses fuzzy PID control method for the control research on the non-linear active hydro-pneumatic suspension system.

The principle of fuzzy control is to fuzzify the input and output parameters, and then establish a fuzzy control rule library based on expert experience. The controller implements fuzzy inference of the control system according to the control rules, and finally defuzzifies the output parameters to obtain specific values.

The PID control algorithm and the principle formula of fuzzy control against PID parameter tuning are:

(15) $$\begin{array}{l} u\left(k\right)=K_{p}e\left(k\right)+K_{i}\int_{i\;=\;0}^{k}e\left(i\right)di+K_{d}\frac{de\left(k\right)}{dk} \end{array}$$

(16) $$\begin{array}{l} K_{p}=K_{p}^{\;\prime }+\Delta K_{p} \end{array}$$

(17) $$\begin{array}{l} K_{i}=K_{i}^{\;\prime }+\Delta K_{i} \end{array}$$

(18) $$\begin{array}{l} K_{d}=K_{d}^{\;\prime }+\Delta K_{d} \end{array}$$

Where, *u*(*k*) is the control system output; $\int_{i\;=\;0}^{k}e\left(i\right)di\;$is the cumulative error; $\frac{de\left(k\right)}{dk}$ is the error rate of change; *K*_*p*_^′^, *K*_*i*_^′^, *K*_*d*_^′^ are the basic values of PID controller parameters; Δ*K*_*p*_, Δ*K*_*i*_, Δ*K*_*d*_ are the correction values of the three parameters of the PID controller; *K*_*p*_, *K*_*i*_, *K*_*d*_ are the final control parameter values of the PID controller.

In the design of the fuzzy PID control algorithm, the error *e* and the error rate of change *e*_*c*_ are selected as the controller input, and the correction values Δ*K*_*p*_, Δ*K*_*i*_, Δ*K*_*d*_ of the three parameters of the PID controller are respectively selected as the fuzzy controller output. After inference and optimization of fuzzy rules, the parameter values required by the PID controller are obtained.

Based on analysis of the suspension working process, seven states are selected as the input and output state variables of the fuzzy controller, which are negative big, negative medium, negative small, zero, positive small, positive middle, and positive big (NB, NM, NS, ZO, PS, PM, PB). Each variable is solved by the triangular membership function.

**Figure 3:**
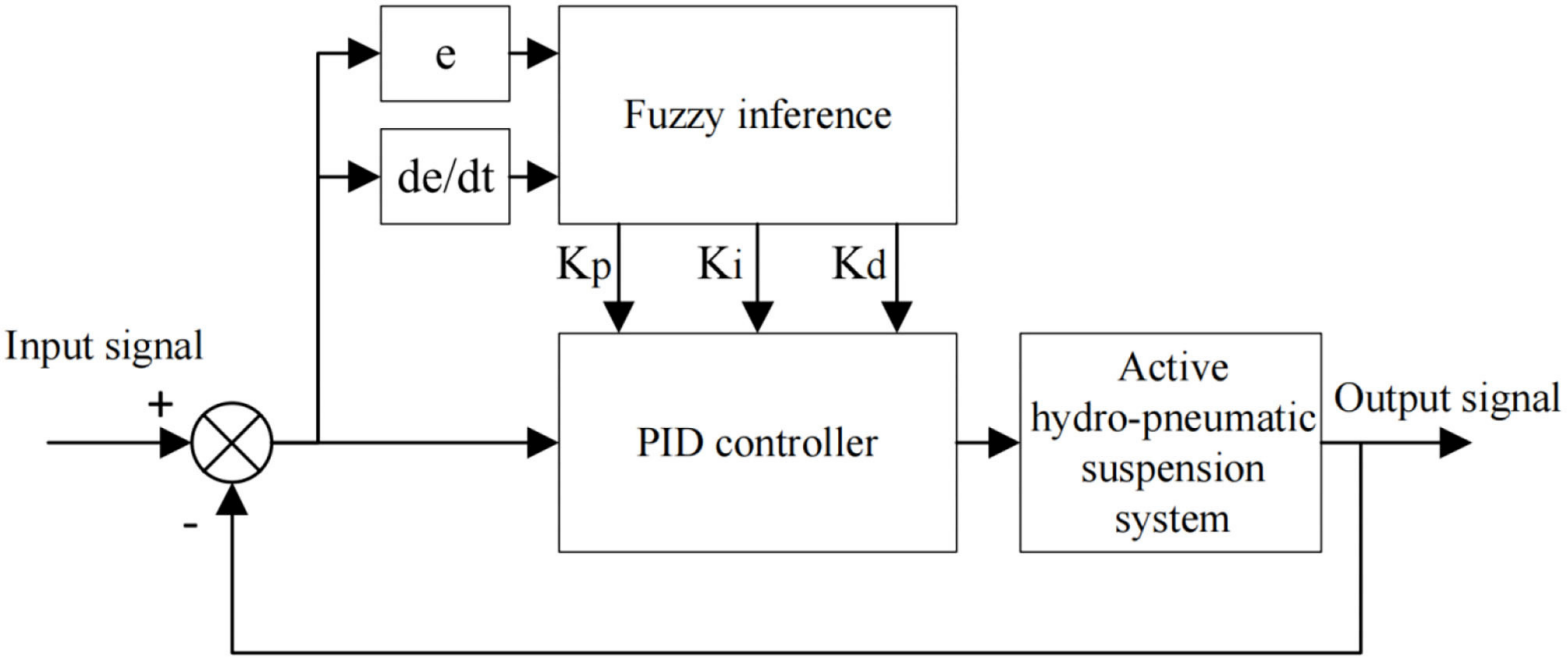
Fuzzy PID control flow chart.

Therefore, based on the above fuzzy controller design, the complete structure principle flow chart of the entire fuzzy PID control active hydro-pneumatic suspension system is shown in Figure 3.

### Genetic Algorithm Optimizes Fuzzy Control Rules

Reasonable fuzzy rules and membership functions can enable the system to achieve more ideal control effect. However, according to the seven fuzzy state variables and the dual-input and three-output in the design, it can be known that there are already 7^5^ fuzzy control rules. For a fuzzy controller that requires expert experience to determine the fuzzy rules, this represents a huge range of values. Therefore, this research uses genetic algorithm to optimize and solve the fuzzy rules required by the system. Genetic algorithm is a computational model that simulates Darwin's biological evolution theory, which searches for the optimal solution of the mathematical model through natural selection and genetic mechanisms.

#### Genetic Coding

When genetic algorithm is used to optimize the fuzzy control rules, considering the number of solutions and the control process running time, the membership function and the domain scope are not optimized. According to the design of fuzzy control input and output, there are five variables in total, and seven fuzzy language values are set in the domain of each variable, so there are 7^5^different fuzzy rules.

According to the fuzzy controller design, the fuzzy language value is digitally coded, and the seven fuzzy language values of PB, PM, PS, ZO, NS, NM, and NB are represented by 1, 2, 3, 4, 5, 6, and 7, respectively.

Therefore, the input variables *e* and *e*_*c*_ are encoded as a 49 × 2 matrix, and the formula is

(19) $$\begin{array}{l} in=\left[\begin{array}{cc} 1 & 1\\ 1 & 2\\ 1 & 3\\ \vdots & \vdots \\ 7 & 5\\ 7 & 6\\ 7 & 7 \end{array}\right] \end{array}$$

According to the design, the fuzzy language value matrix of the output values Δ*K*_*P*_, Δ*K*_*I*_, Δ*K*_*D*_ is the result required for the genetic algorithm solution. The fuzzy language value matrix of the output variable is denoted by *X*, which is a 49 × 3 matrix. Therefore, the digitized matrix of fuzzy rules is

(20) $$\begin{array}{l} rule=\left[in,\;X\right] \end{array}$$

The matrix row vector represents the fuzzy control rule. According to the coding requirements of genetic algorithm, re-arrange the fuzzy rules of the three output variables into a row of vectors in order, which is the length of the individual chromosomes in the population, and its length is 7 × 7 × 3.

#### Fitness Function

Fitness refers to the ability of individuals to adapt to the environment during the population evolution. In the process of biological evolution, individuals with low fitness have a small survival probability, and individuals with high fitness have a high survival probability. After retaining individuals with high fitness, better individuals can be created through multiple iterations.

Therefore, the fitness function is the key function for genetic algorithm optimization. The fitness function in the genetic algorithm should meet the requirements of maximization, and the objective function aims to find the minimum value. In the genetic algorithm, time multiplied by absolute error integral criterion (ITAE) is selected as the optimized performance index, and *e* is selected as the acceleration. Under larger fitness value, time multiplied by the absolute error integral value is smaller, indicating that the control effect is fine. Its fitness function is expressed as

(21) $$\begin{array}{l} J\left(ITAE\right)=\frac{1}{\int_{0}^{\infty }t|e\left(t\right)|dt} \end{array}$$

#### Genetic Operators

The general steps of genetic algorithm optimization:

1) Initialize the population;
2) Calculate fitness value;
3) Selection, crossover, mutation;
4) Generate a new population.

According to the genetic algorithm optimization process, after setting the genetic algebra, the population will repeat the above general optimization steps, and iteratively generate new populations with higher individual fitness.

The purpose of “selection” is to select individuals with better fitness from individuals in the population through operator action to directly inherit them to the next generation. According to experience, continue to test and select the best parameters. The selection probability is set to 0.9 in this paper.

The “crossover” process is to randomly select two chromosomes, then randomly select the positions of the chromosomes for exchange, and the selected position length of the two chromosomes is the same. The crossover probability is set to 0.8 in this paper.

The “mutation” process is to replace the value of a certain position in the chromosome with a random number between 1~7. According to experience, continue to test and select the best parameters. The mutation probability is set to 0.08 in this paper.

## Control System Simulation Analysis and Experiment

In view of the designed active hydro-pneumatic suspension control system model, the system simulation model is built through the simulink simulation module in MATLAB software.

### Establishment of Random Road Model

In this simulation, the filtered white noise method was used to reconstruct the road time domain model that meets the established power spectral density, that is, the elevation change of the road surface is abstracted into random white noise that satisfies certain conditions, and then transformed to obtain the time domain model expression of random road unevenness:

(22) $$\begin{array}{l} \dot{w}\left(t\right)=2\pi n_{0}\sqrt{G_{w}\left(n_{0}\right)u}q\left(t\right) \end{array}$$

Where, q(t) represents the band-limited white noise with a mean value of 0 and a covariance of 1*m*^2^/*s*; *G*_*w*_(*n*_0_) is the road unevenness coefficient, which represents the road power spectral density value under the reference spatial frequency *n*_0_, *m*^2^/*m*^−1^; u is the vehicle speed, *m*/*s*; *n*_0_ is the reference spatial frequency, taking *n*_0_= 0.1*m*^−1^; ẇ(*t*) represents the vertical speed under road unevenness, m/s.

Compared with the standard road surface, the mean square error value of the random road time domain signal obtained by Equation (22) has a big error. To truly reflect the road surface working condition at low frequency, the road space cut-off frequency *n*_*d*0_ and the parameters δ, γ are introduced and incorporated into Equation (23). The random elevation mean square error value of the modified time domain model is approximately equal to the specified value in the standard pavement grade, and the power spectral density value is also in good agreement. The random road unevenness time-domain model of the sprayer under common speed conditions and different grades is obtained as

(23) $$\begin{array}{l} \dot{w}\left(t\right)=-2\pi n_{d0}u\delta w\left(t\right)+2\pi n_{0}\gamma \sqrt{G_{w}\left(n_{0}\right)u}q\left(t\right) \end{array}$$

Where, w(t) is the road displacement input, m; δ = 1.61, γ = 7.07, *n*_*d*0_ is the road cutoff spatial frequency, and the value can be around 0.011*m*^−1^ to generate the time domain road input.

**Figure 4:**
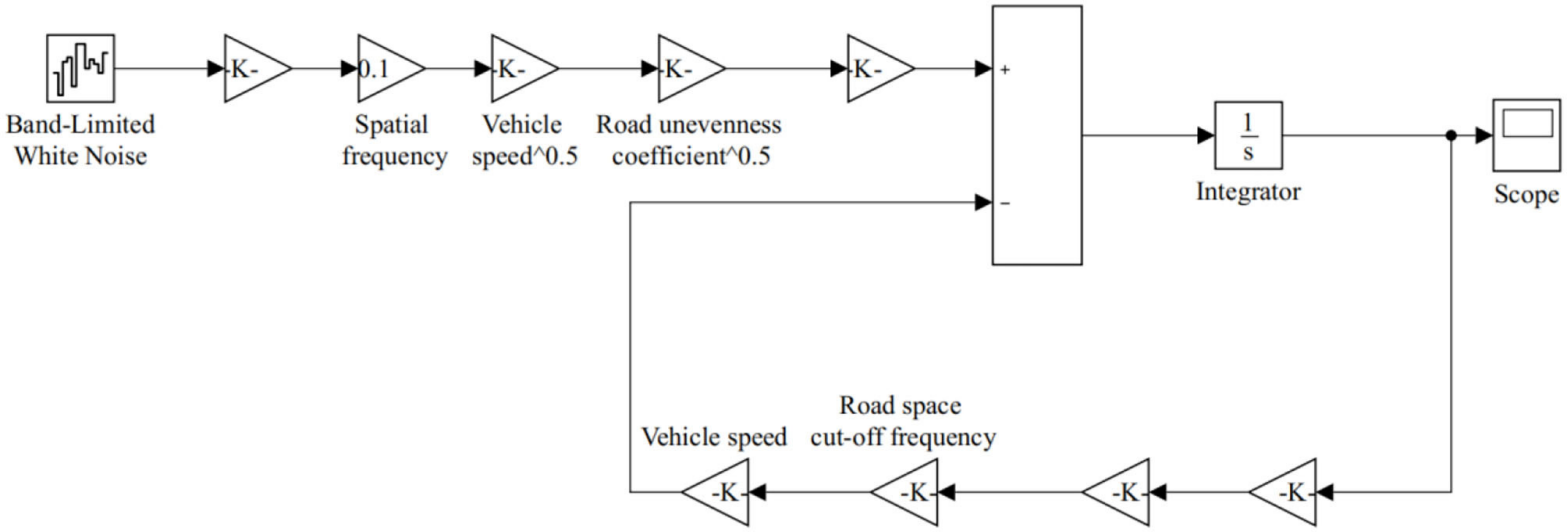
Road excitation model.

Based on the definition of the random road power spectrum in the national standard, in view of the description relationship between time domain and frequency domain, with road grade E as the criterion, set the road unevenness coefficient $G_{w}\left(n_{0}\right)=1024\;\times 10^{-6}m^{3}$, set the vehicle speed to 10 Km/h, and use Matlab/Simulink to establish a random road simulation model for the time domain model described by Equation (23), as shown in Figure 4.

### Controller Parameter Solution and Design

In view of the designed active hydro-pneumatic suspension control system model, the fuzzy control rules are optimized by the genetic algorithm through MATLAB software programming. The algorithm operator optimizes the processing of individual chromosomes in the population, makes the population continue to iterate to the maximum genetic algebra, and then outputs the optimal solution.

The Fuzzy-PID control input of the active hydro-pneumatic suspension is the suspension vertical acceleration deviation *e* and the vertical acceleration deviation change *e*_*c*_. The correction values *K*_*P*_, *K*_*I*_, *K*_*D*_ of the three PID controller parameters are selected as the output of the fuzzy controller. In the controller, the domains of *e* and *e*_*c*_ are both [−6, 6], and the domains of *K*_*P*_, *K*_*I*_, *K*_*D*_ are all [−10, 10]. The two inputs *e*, *e*_*c*_ and the three outputs *K*_*P*_, *K*_*I*_, *K*_*D*_ are all defined as seven fuzzy subsets: PB (positive big), PM (positive middle), PS (positive small), ZO (zero), NS (negative small), NM (of negative middle), and NB (negative big). The three output variables and the two input variables all adopt triangular membership functions.

The fuzzy PID control model is built through MATLAB/simulink and co-simulated with the active hydro-pneumatic suspension model using the random road model as the road surface excitation.

The goal of control research in this paper is to control the vertical acceleration of the vehicle body. The difference between the preset value and the true value of the controlled object is used as input in calculation, and the solenoid valve is controlled to achieve control. A fuzzy PID control model is built in Matlab/Simulink and co-simulated with the active hydro-pneumatic suspension model using random road model as the road excitation.

### Simulation Results and Experimental Verification

**Figure 5:**
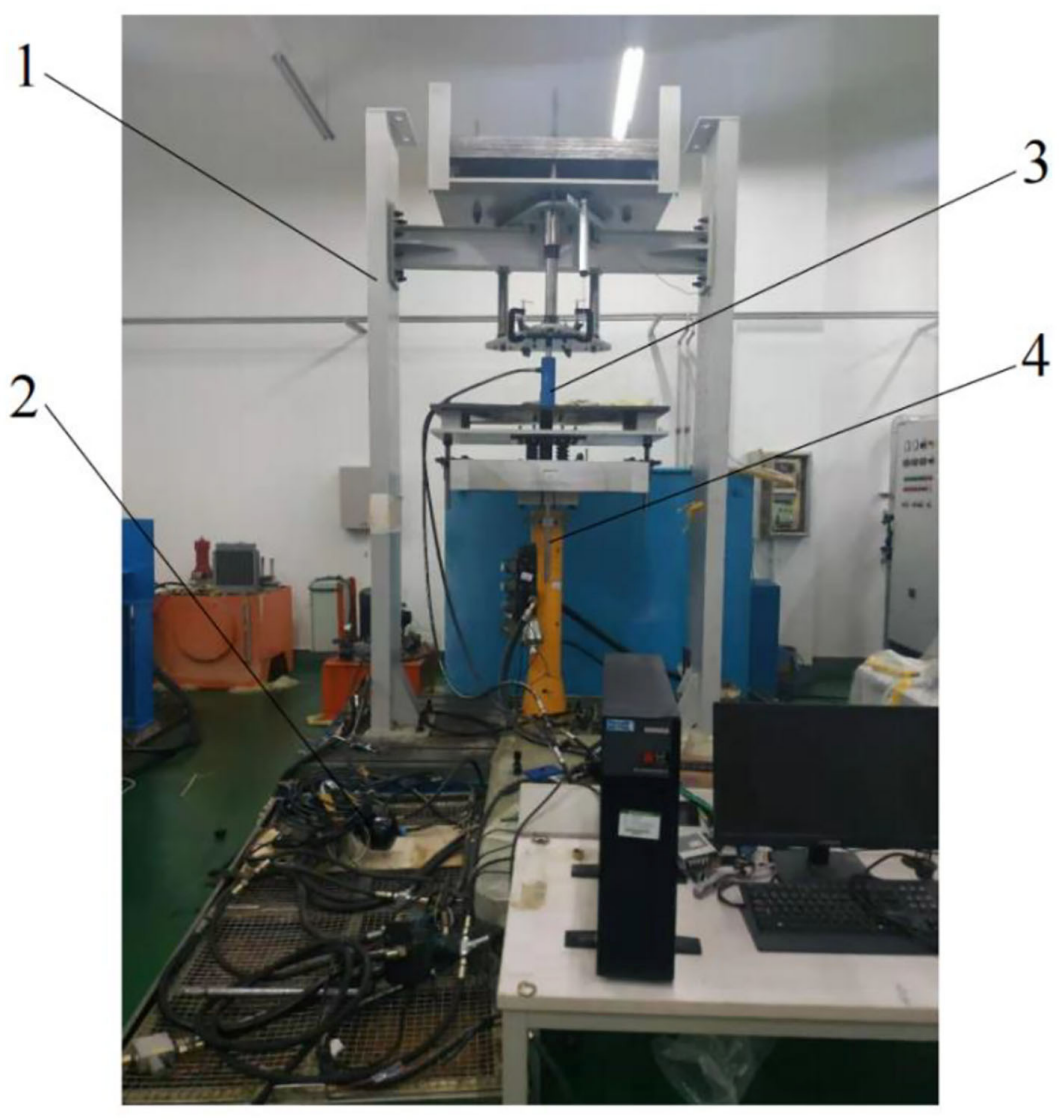
Active hydro-pneumatic suspension test bench. 1. Vibration test bench 2. Accumulator 3. Hydraulic cylinder 4. Exciting hydraulic cylinder.

The indoor experimental system of active hydro-pneumatic suspension is shown in Figure 5. In the experiment, oil charging and discharging control is completed by the oil circuit integrating the proportional flow valve and the electromagnetic reversing valve. The proportional flow valve is an isolating balance valve (model iQ203311) produced by Teknocraft, which adjusts the flow rate by adjusting the input voltage to change the valve opening. The reversing valve adopts SNACE's 4V330C-10 three-position five-way solenoid valve, which implements oil charging and discharging function by regulating valve port switch via high and low levels. The controller adopts the NI PXIe-8135 real-time control system. Its advantage is that the control algorithm code edited in Matlab can be directly converted, downloaded and implemented. The control system is equipped with up to 32 analog input and output to facilitate actual verification of the control strategy. The acceleration sensor is used to collect the acceleration *ẍ*_*s*_, *ẍ*_*u*_ of the sprung mass and the unsprung mass, respectively. The displacement sensor is used to collect the displacement *x*_*s*_ − *x*_*u*_ between the sprung mass and the unsprung mass, and the displacement *x*_*u*_ − *x*_*r*_ between the unsprung mass and the “ground.” Then, the movement speed *ẋ*_*s*_ and *ẋ*_*u*_ of the sprung mass and the unsprung mass are calculated through calculus operation or estimation of the state observer. Based on this, all state variables in the working process can be measured in real time or obtained through further calculations.

#### Simulation Results

The relevant parameters of the active hydro-pneumatic suspension experimental system are shown in Table 1.

**Table 1:** The main parameters of the active hydro-pneumatic suspension experimental system.

**Parameter**	**Value/Unit**	**Parameter**	**Value/Unit**
Sprung mass m_1_	3875 kg	Oil supply pressure	10 mpa
Unsprung mass m_2_	350 kg	Unloading oil pressure	0.1 mpa
Initial oil storage volume of cylinder v_10_	1.62 × 10^−3^ m	Solenoid valve proportional coefficient ku	2.85 × 10^−4^
Cylinder action area A_1_	6.34 × 10^−3^ m^2^	Tire equivalent stiffness Kt	650
Orifice discharge coefficient c_d_	0.7	Tire equivalent damping Ct	6.6
Effective elastic modulus of oil β_e_	1200 mpa	Damping hold flow area A_K_	2.67 × 10^−4^ m^2^
Oil density	890	Initial gas volume V_go_	4.195 × 10^−3^ m^3^

*The optimized fuzzy rule table is shown in Table 2*.

**Table 2:** Fuzzy PID control rule table.

**e**	****e****_******c******_						
	**NB**	**NM**	**NS**	**ZO**	**PS**	**PM**	**PB**
NB	PB/PB/NS	NB/PB/PS	PB/ZO/PM	NM/NS/NM	NM/NM/PM	NB/NM/NB	PB/PB/NM
NM	PM/ZO/PB	NS/ZO/PB	PB/NS/NS	ZO/NM/PB	PB/NM/NS	NS/NS/NB	PB/PS/PM
NS	ZO/NB/NS	NM/NS/PM	ZO/NM/ZO	PB/PM/PS	NB/PM/ZO	PS/NS/PM	NS/NM/PM
ZO	NB/PS/PB	ZO/PM/PM	NM/NM/NM	NS/PB/PB	NS/PB/NM	NS/PB/PB	NM/NM/NS
PS	PB/NS/PB	NM/PM/PB	NB/NB/NS	PS/NB/PM	NB/ZO/ZO	PS/NS/PM	PB/PS/NB
PM	PB/PM/ZO	PB/PM/NS	NM/NS/PB	PM/PB/NS	NB/NB/NS	PB/PS/NM	PB/NB/NS
PB	ZO/ZO/NB	NM/PB/NB	NB/PS/PB	PM/NB/PB	NS/PM/PM	PS/PS/NM	PB/NS/PB

**Figure 6:**
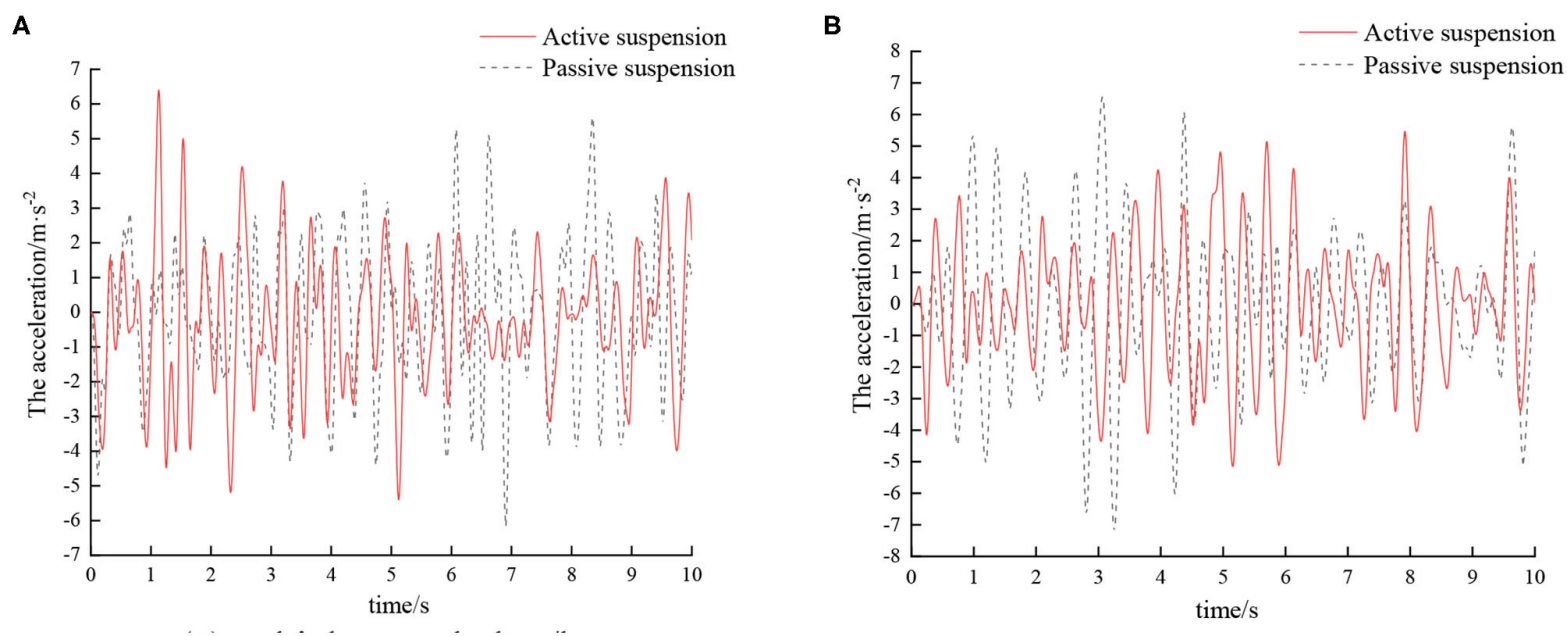
Time domain response curve of sprayers at different speeds on D-grade roads. **(A)** Vehicle speed 5 km/h. **(B)** Vehicle speed 8 km/h.

**Figure 7:**
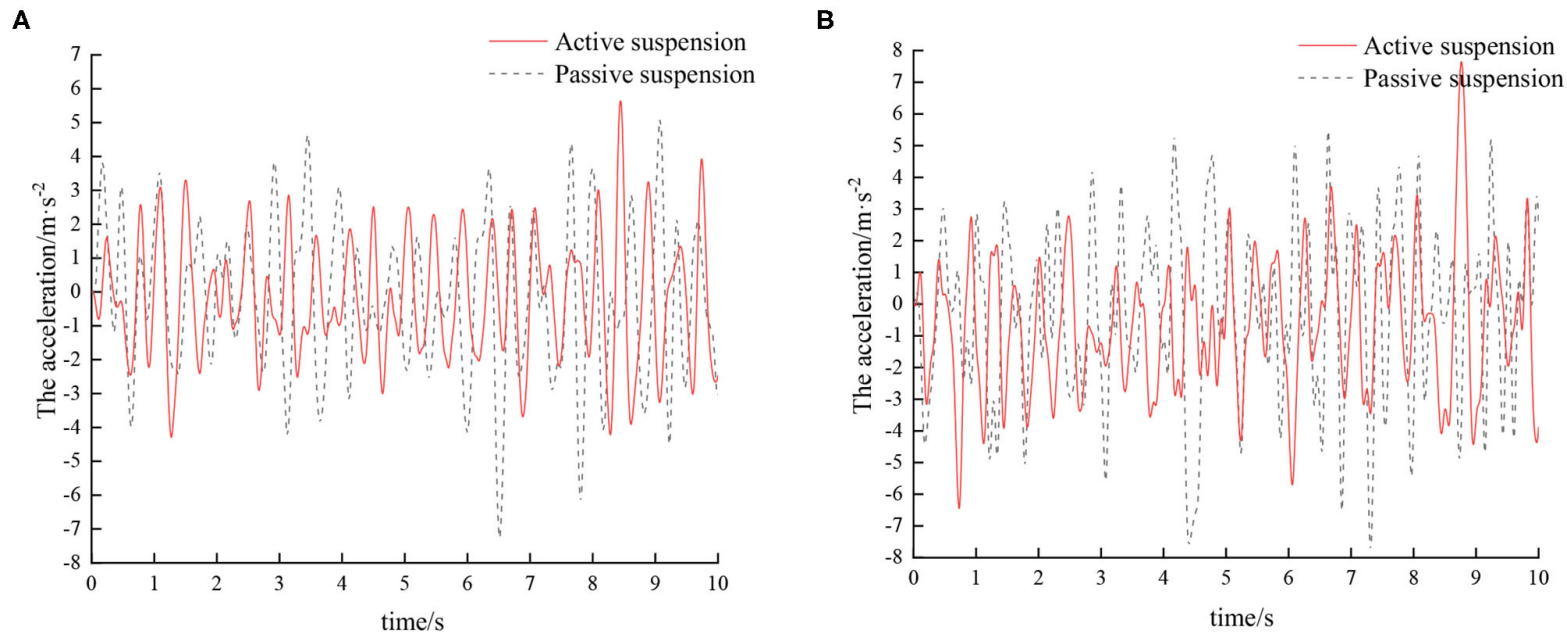
Time domain response curve of sprayers at different speeds on E-grade road. **(A)** Vehicle speed 5 km/h. **(B)** Vehicle speed 8 km/h.

The optimized fuzzy rules are imported into the fuzzy controller, and the active hydro-pneumatic suspension system is simulated, with simulation results shown in Figures 6, 7.

According to the vibration acceleration signal obtained by the simulation, the root mean square acceleration value of the sprayer under different vehicle speeds on the D-grade road surface after vibration damping by different suspensions is shown in Table 3.

**Table 3:** Comparison of root mean square acceleration values of sprayers at different speeds on D-grade road.

**Type**	**Root mean square acceleration value (m/s**^****2****^**)**	
	**5 km/h**	**8 km/h**
Passive suspension	1.989	2.354
Active suspension	1.763	2.063
Performance improvement	11.36%	12.36%

According to the vibration acceleration signal obtained by the simulation, the root mean square acceleration value of the sprayer under different vehicle speeds on E-grade road surface after vibration damping by different suspensions is shown in Table 4.

**Table 4:** Comparison of root mean square acceleration values of sprayers at different speeds on E-grade road.

**Type**	**Root mean square acceleration value (m/s**^****2****^**)**	
	**5 km/h**	**8 km/h**
Passive suspension	2.120	2.518
Active suspension	1.839	2.143
Performance improvement	13.25%	14.89%

Combining Tables 3, 4, it can be seen that compared with the traditional passive suspension, the vibration damping performance of the active suspension has certain improvement. On D-grade roads, as the speed increases, active suspension has superior damping effect than traditional passive suspension, and the corresponding root mean square values of vibration acceleration of the vehicle body are reduced by 11.36 and 12.36%, respectively. On E-grade roads, with the increase of speed, the corresponding root mean square values of vibration acceleration of the vehicle body are reduced by 13.25 and 14.89%, respectively. With the increase of road surface and vehicle speed, the active suspension has superior damping effect than the passive suspension. This is mainly because as the speed increases, the road surface excitation frequency gradually increases. That is, the vibration transmitted to the wheels by the road surface is more intense. As a result, the vehicle body vibration amplitude also increases. The active suspension adjusts the suspension stiffness and damping through this control system, so that the vibration damping performance of the sprayer is improved. Hence, from an overall point of view, compared to passive suspension, active suspension can better improve the damping characteristics of the sprayer chassis.

#### Experimental Verification

**Figure 8:**
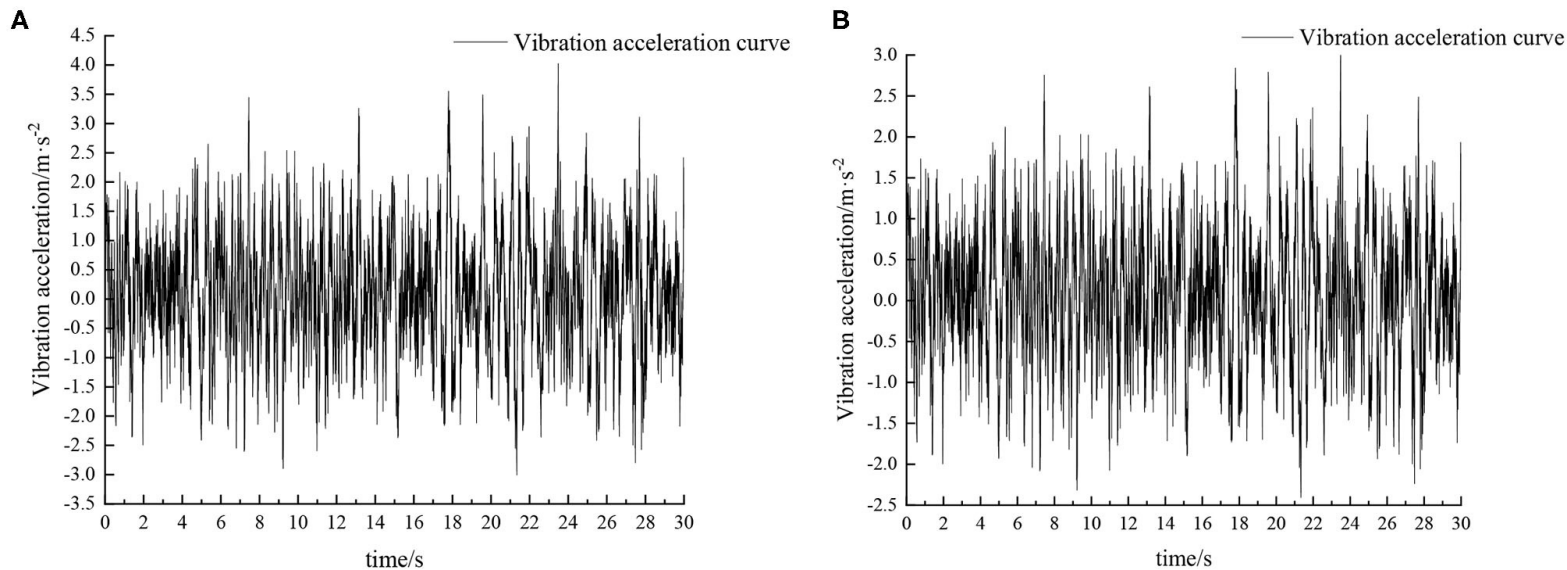
Time domain response curve when driving at 5 km/h on field road. **(A)** Passive suspension. **(B)** Active suspension.

**Figure 9:**
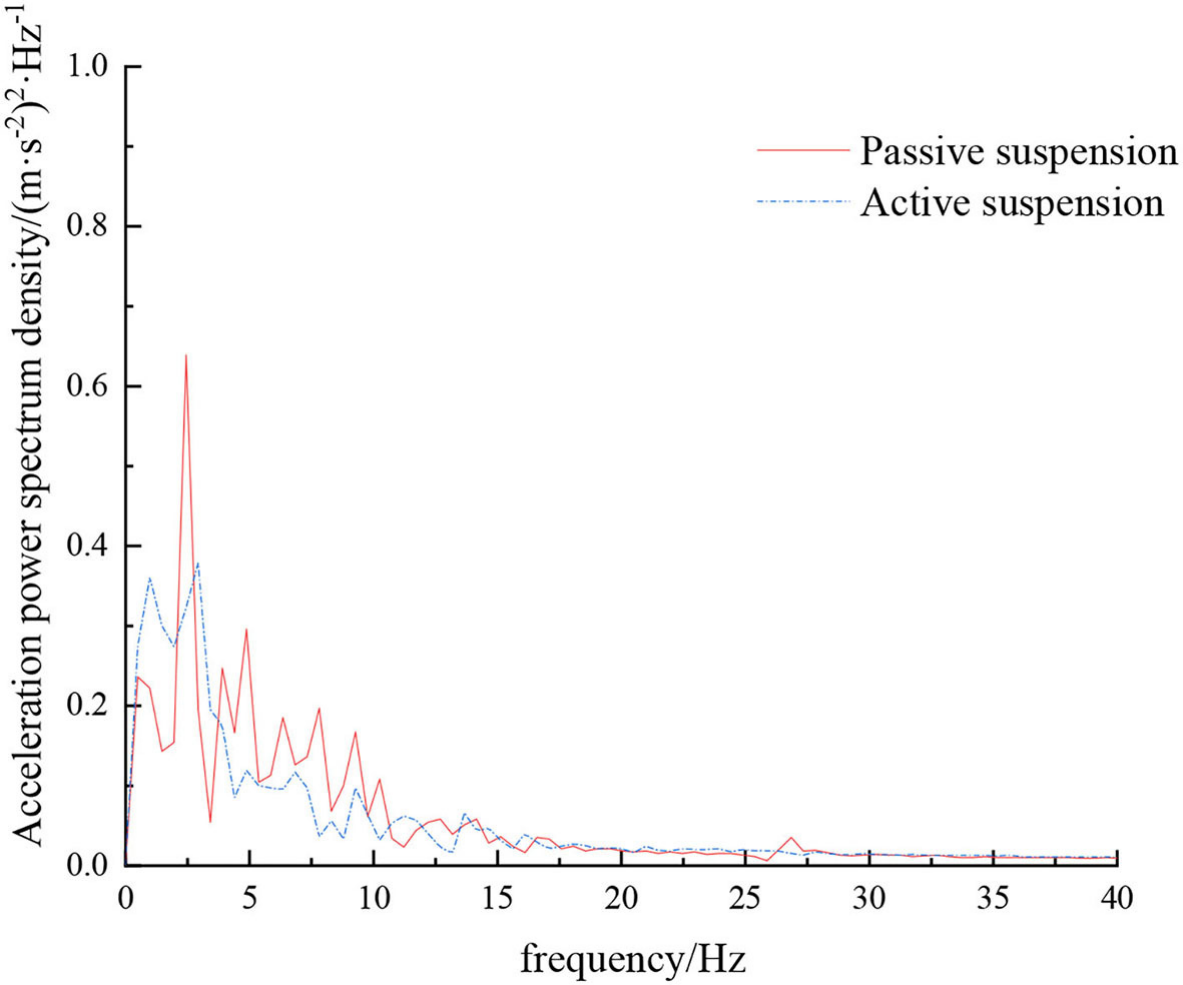
Frequency domain response curve when driving at 5 km/h on field road.

**Figure 10:**
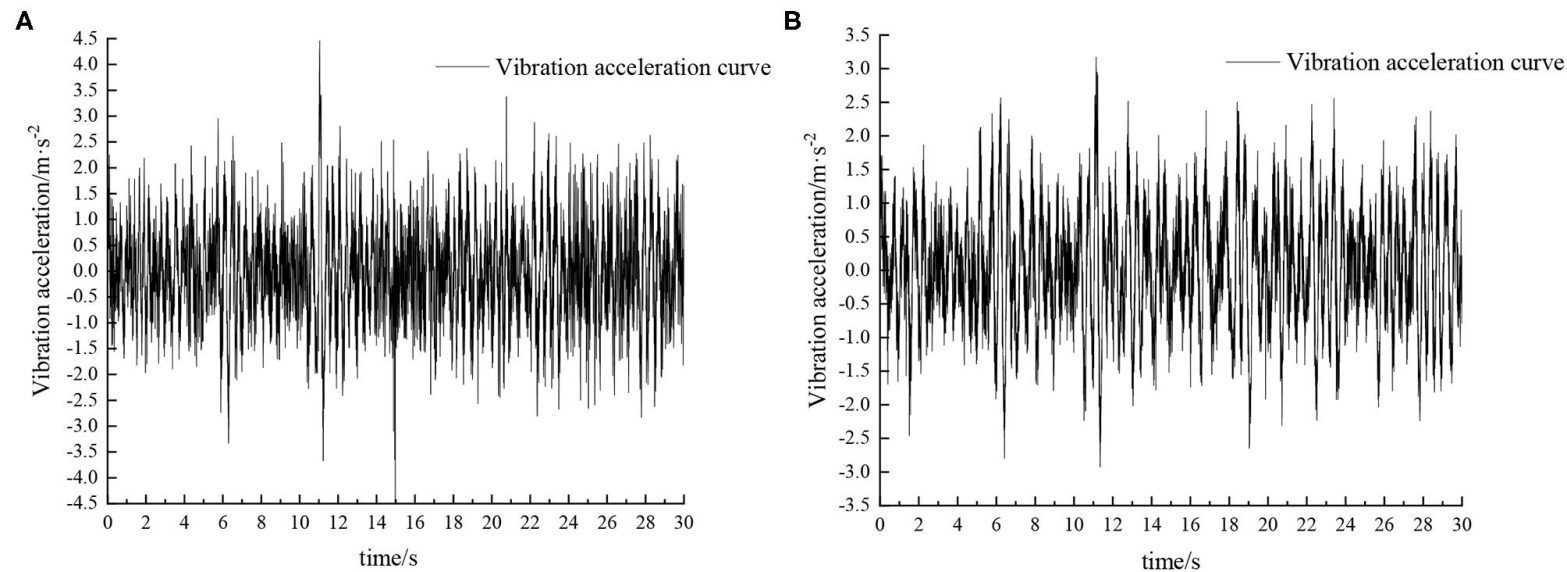
Time domain response curve when driving at 8 km/h on field road. **(A)** Passive suspension. **(B)** Active suspension.

**Figure 11:**
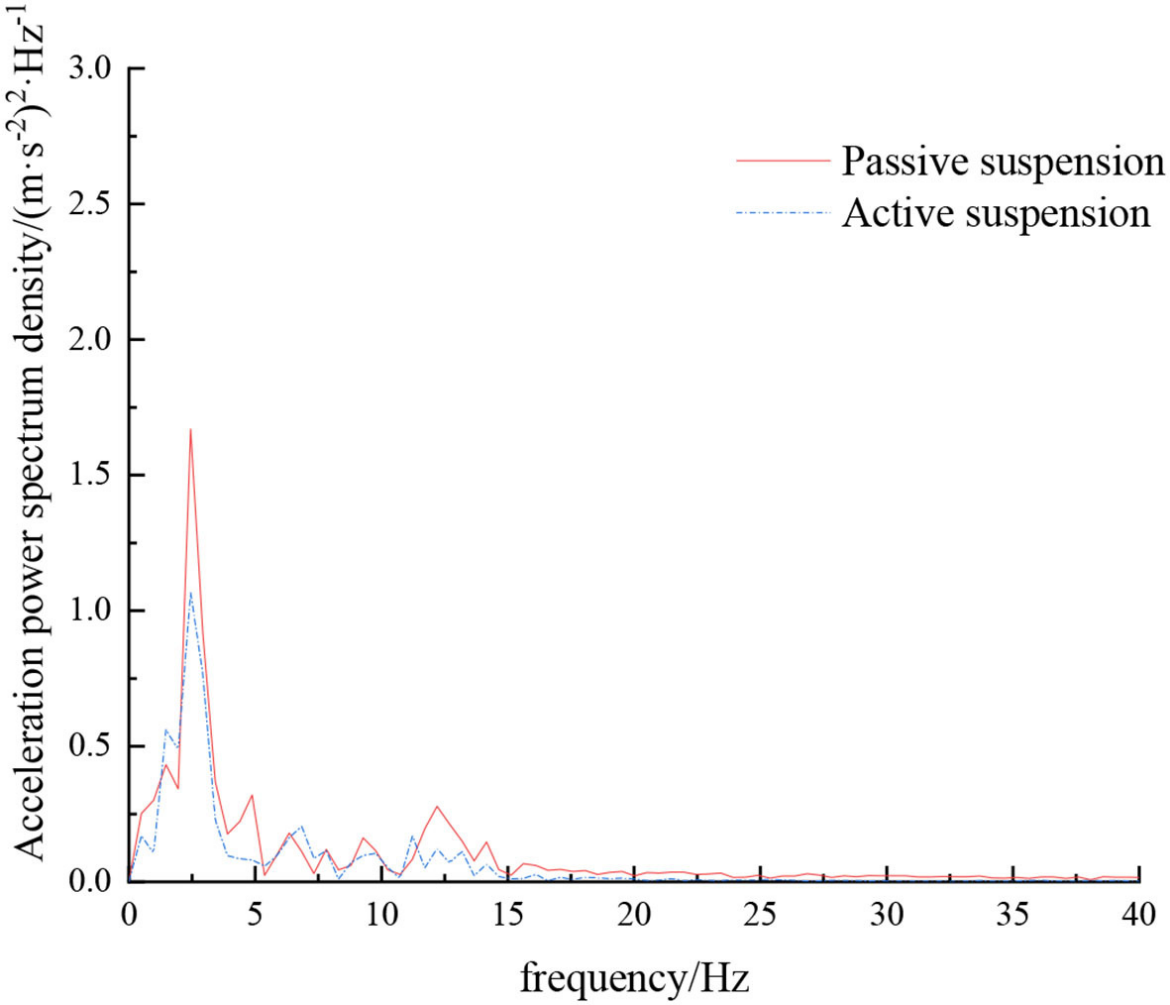
Frequency domain response curve when driving at 8 km/h on field road.

Experiments were carried out by simulating field roads and sand-gravel roads with the vibration platform of the active hydro-pneumatic suspension test bench. The experimental results are shown in Figures 8–11.

When the sprayer is simulated to run on the field road at different speeds through the experimental platform, the time domain response curve of vehicle body vibration acceleration measured when the sprayer is running at 5 km/h is shown in Figure 8. Where, the root mean square acceleration values of the passive and active suspensions are 1.080 and 0.847 m/s^2^, respectively. The time domain response curve of vehicle body vibration acceleration measured when the sprayer is running at 8 km/h is shown in Figure 10. Where, the root mean square acceleration values of passive and active suspensions are 1.412, 1.125 m/s^2^, respectively. By comparing the root mean square value of the vehicle body vibration acceleration, it can be known that the root mean square value of the vehicle body vibration acceleration is greatly reduced after vibration damping of the active suspension when the sprayer is simulated to drive on field road at different speeds, indicating that active suspension has a good damping effect. According to the test results, the frequency domain curve of the sprayer is obtained by performing FFT transformation on the vibration acceleration change curve, as shown in Figures 9, 11. It can be seen from Figure 9 that the peak value of the vibration acceleration power spectrum density of the passive and active suspensions of the sprayer is mainly concentrated in 0~5 Hz. Where, the passive suspension has a peak value of 0.766 (m/s^2^)^2^/Hz, and the active suspension has a peak value of 0.479 (m/s^2^)^2^/Hz. As can be seen from Figure 11, the peak value of the vibration acceleration power spectrum density of the passive and active suspensions of the sprayer is mainly concentrated in 0~5 Hz. Where, the passive suspension has a peak value of 1.663 (m/s^2^)^2^/Hz, and the active suspension has a peak value of 1.049(m/s^2^)^2^/Hz. From this, it can be seen that compared with the corresponding reduction in peak vibration acceleration of the passive suspension, there is greater corresponding frequency reduction in peak value of the active suspension, indicating that the active suspension has good vibration isolation performance.

**Figure 12:**
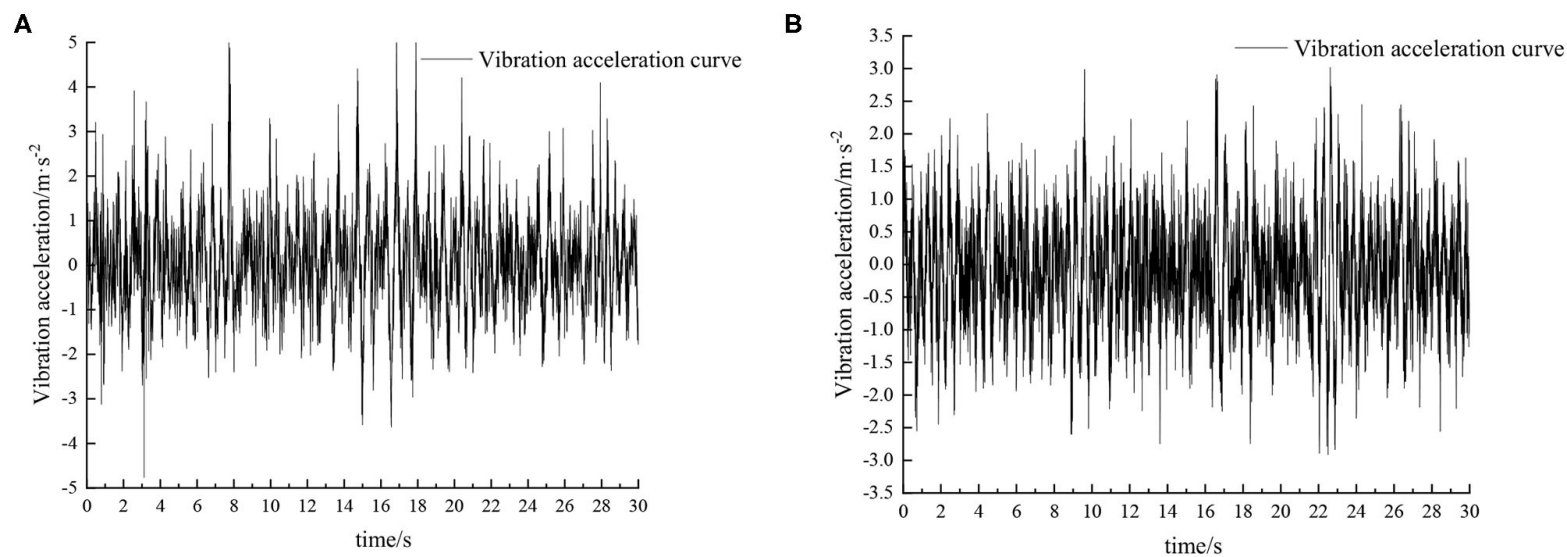
Time domain response curve when driving at 5 km/h on sand-gravel road. **(A)** Passive suspension. **(B)** Active suspension.

**Figure 13:**
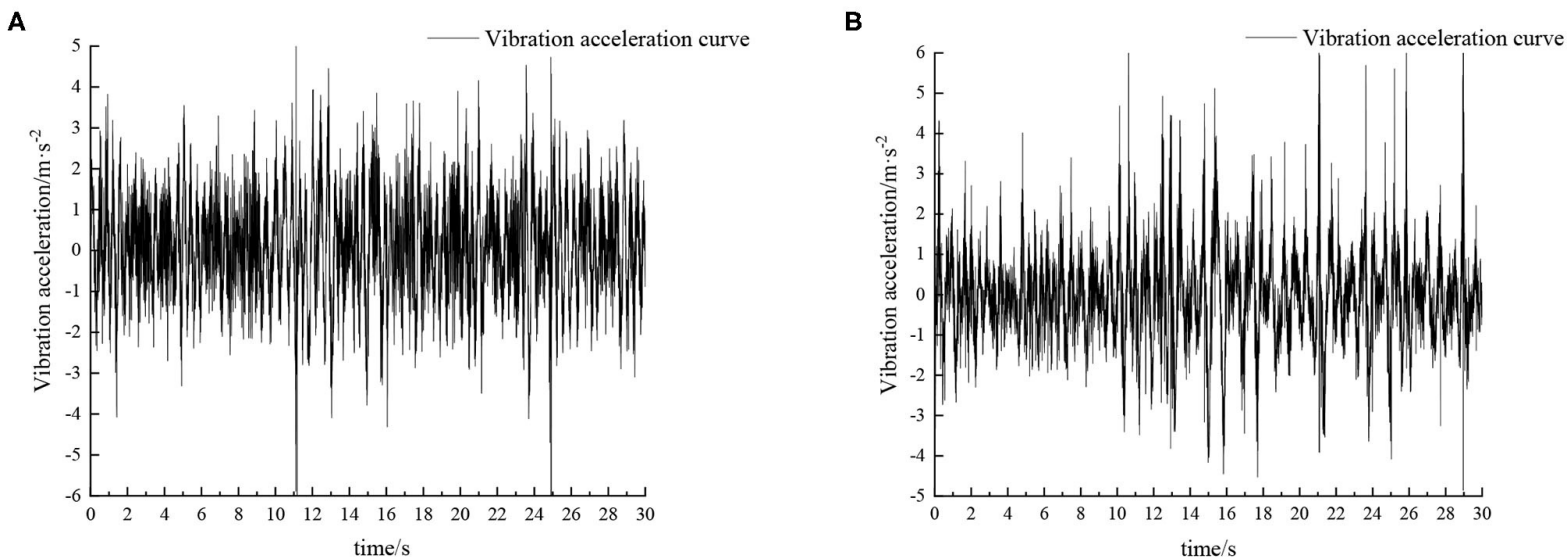
Time domain response curve when driving at 8 km/h on sand-gravel road. **(A)** Passive suspension. **(B)** Active suspension.

**Figure 14:**
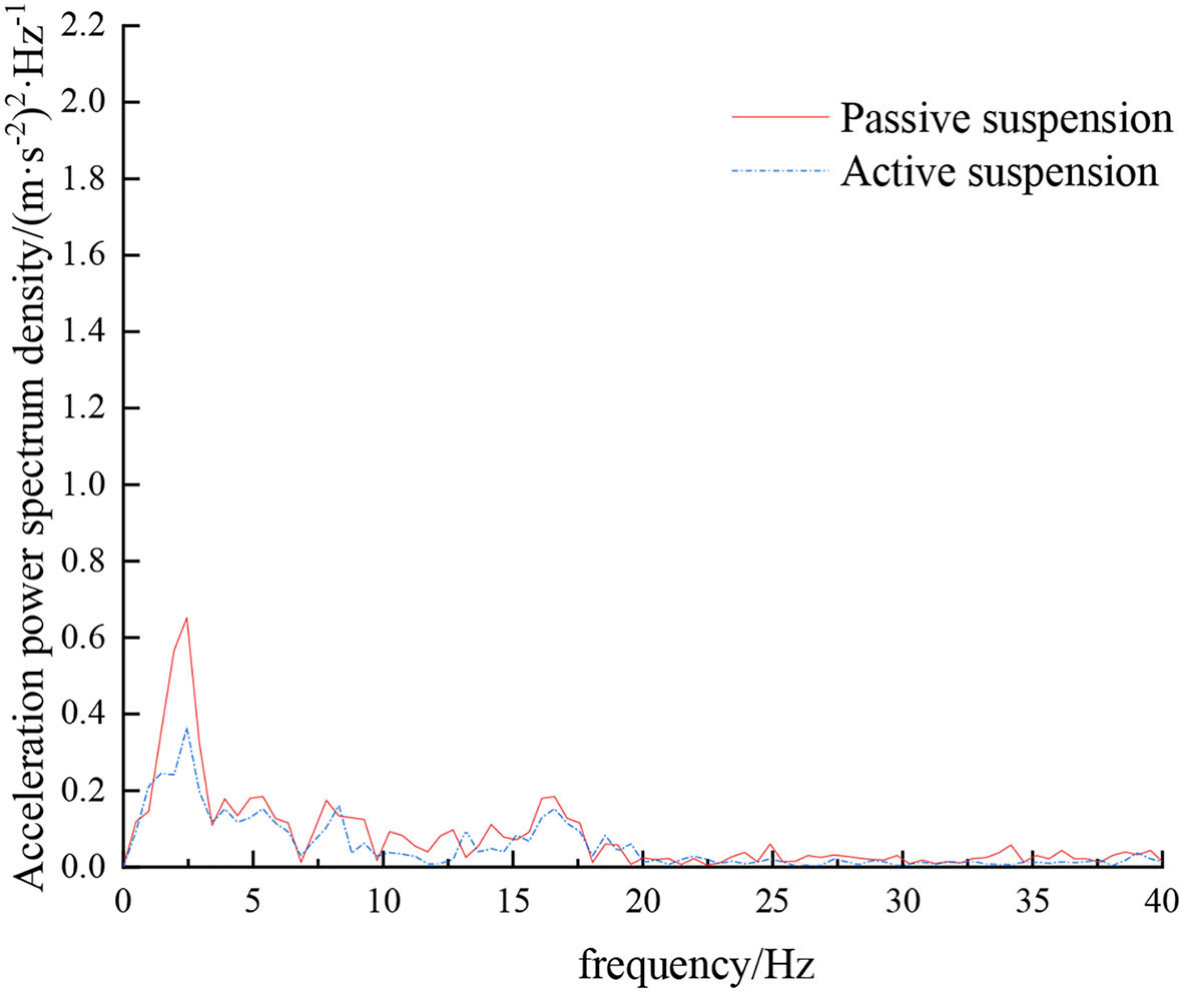
Frequency domain response curve when driving at 5 km/h on sand-gravel road.

**Figure 15:**
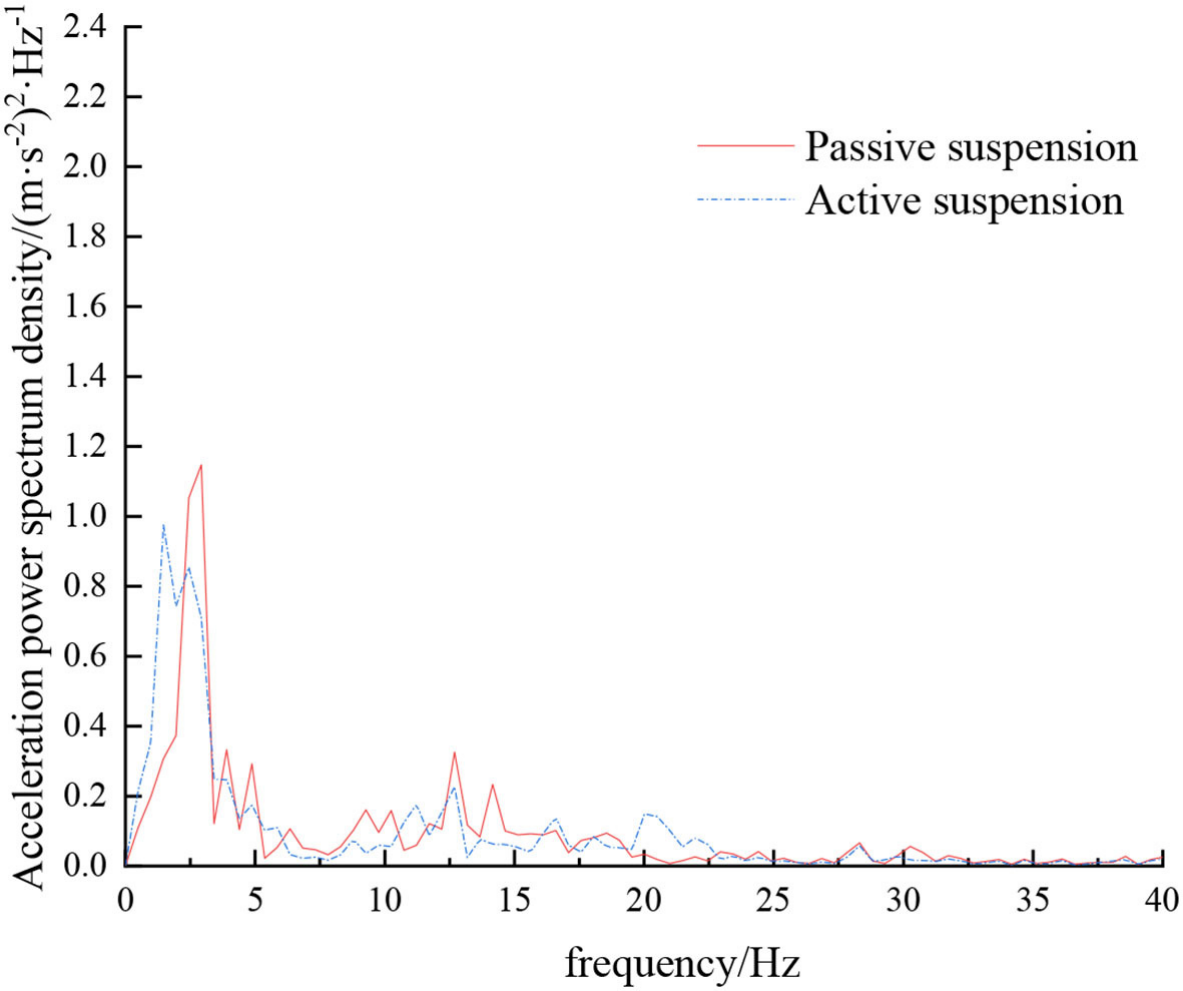
Frequency domain response curve when driving at 8 km/h on sand-gravel road.

When the sprayer is simulated to run on the sand-gravel road at different speeds through the experimental platform, the time domain response curve of vehicle body vibration acceleration measured when the sprayer is running at 5 km/h is shown in Figure 12. Where, the root mean square acceleration values of the passive and active suspensions are 1.149 and 0.891 m/s^2^, respectively. The time domain response curve of vehicle body vibration acceleration measured when the sprayer is running at 8 km/h is shown in Figure 13. Where, the root mean square acceleration values of passive and active suspensions are 1.572, 1.229 m/s^2^, respectively. By comparing the root mean square value of the vehicle body vibration acceleration, it can be known that the root mean square value of the vehicle body vibration acceleration is greatly reduced after vibration damping of the active suspension when the sprayer is simulated to drive on sand-gravel road at different speeds, indicating that active suspension has a good damping effect. According to the test results, the frequency domain curve of the sprayer is obtained by performing FFT transformation on the vibration acceleration change curve, as shown in Figures 14, 15. It can be seen from Figure 14 that the peak value of the vibration acceleration power spectrum density of the passive and active suspensions of the sprayer is mainly concentrated in 0~5 Hz. Where, the passive suspension has a peak value of 0.652 (m/s^2^)^2^/Hz, and the active suspension has a peak value of 0.364 (m/s^2^)^2^/Hz. As can be seen from Figure 15, the peak value of the vibration acceleration power spectrum density of the passive and active suspensions of the sprayer is mainly concentrated in 0~5 Hz. Where, the passive suspension has a peak value of 1.147 (m/s^2^)^2^/Hz, and the active suspension has a peak value of 0.976(m/s^2^)^2^/Hz. From this, it can be seen that compared with the corresponding reduction in peak vibration acceleration of the passive suspension, there is greater corresponding frequency reduction in peak value of the active suspension, indicating that the active suspension has good vibration isolation performance.

It can be seen from Table 5, Table 6 that as the sprayer operating speed increases, the root mean square value of the vertical acceleration of the sprayer suspension gradually increases. The main reason is that with the increase of speed, the frequency of road excitation gradually increases. Therefore, appropriate operating speed should be chosen during the sprayer operation process to not only ensure the riding comfort of the sprayer during the operation, but also reduce the spray boom vibration and improve the sprayer operation effect. When the sprayer runs on the field road and sand-gravel road at the same speed, the root mean square value of vibration acceleration is higher on the sand-gravel road than on the field road. This is mainly because the sand-gravel road has higher hardness than the field road. With higher unevenness and road excitation, the sprayer vibration amplitude is also higher. On the field road, with the increase of speed, the active suspension has superior damping effect than the traditional passive suspension, and the corresponding root mean square value of vehicle body vibration acceleration is reduced by 21.57 and 20.33%, respectively. On sand-gravel road, with the increase of speed, the corresponding root mean square value of vehicle body vibration acceleration decreases by 22.45 and 21.82%, respectively. Moreover, with the increase of vehicle speed and road surface level, there is increasingly larger difference between the root mean square acceleration values of passive suspension and active suspension, and active suspension has a better damping effect than passive suspension. Therefore, compared with passive suspension, active suspension is more suitable to operate on complex road conditions.

**Table 5:** Different root mean square values of vibration acceleration when driving under field road excitation.

**Type**	**Root mean square acceleration value (m/s**^****2****^**)**	
	**5 km/h**	**8 km/h**
Passive suspension	1.080	1.412
Active suspension	0.847	1.125
Performance improvement	21.57%	20.33%

**Table 6:** Different root mean square values of vibration acceleration when driving under sand-gravel road excitation.

**Type**	**Root mean square acceleration value (m/s**^****2****^**)**	
	**5 km/h**	**8 km/h**
Passive suspension	1.149	1.572
Active suspension	0.891	1.229
Performance improvement	22.45%	21.82%

## Conclusion

In view of the suspension system of high ground clearance self-propelled sprayer, this study designs a timely started hydro-pneumatic suspension based on fuzzy PID control strategy optimized by genetic algorithm, performs modeling simulation, and carries out indoor bench experiment verification, with the conclusions drawn as follows:

(1) During system simulation, the sprayer is driving on D-grade road. As the speed increases, compared with the traditional passive suspension, the root mean square value of the corresponding vehicle body vibration acceleration of the active suspension is reduced by 11.36, 12.36%, respectively. On E-grade road, as the speed increases, the root mean square value of corresponding vehicle body vibration acceleration decreases by 13.25 and 14.89%, respectively.
(2) During the experimental verification, under field road excitation, when the sprayer traveled at 5 km/h, the root mean square acceleration values of the passive and active suspensions were 1.080 and 0.847 m/s^2^, respectively; when the sprayer traveled at 8 km/h, the root mean square acceleration values of the passive and active suspensions were 1.412 and 1.125 m/s^2^, respectively, with the root mean square values of vibration acceleration reduced by 21.57 and 20.33%, respectively. Under sand-gravel road condition, when the sprayer traveled at 5 km/h, the root mean square acceleration values of the passive and active suspensions were 1.149 and 0.891 m/s^2^, respectively; when the sprayer traveled at 8 km/h, the root-mean-square acceleration values of the passive and active suspensions were 1.572 and 1.229 m/s^2^, respectively, with the root mean square values of vibration acceleration reduced by 22.45 and 21.82%, respectively. Experiments on field roads and sand-gravel roads under excitation conditions have shown that the active air suspension has a significant damping effect, and as the vehicle speed and road grade increase, the active suspension has a better damping effect than the passive suspension. It suggests that the proposed fuzzy PID control strategy based on genetic algorithm optimization can effectively suppress severe jitter of the vehicle body, which in turn improves operation reliability, safety and ride comfort.

## Data Availability Statement

The original contributions presented in the study are included in the article/supplementary material, further inquiries can be directed to the corresponding author.

## Author Contributions

CQ: conceptualization, methodology, experimental ideas, software, writing and editing, and writing–original draft. HW: financial support for publishing experiments, reviewed and revised the draft, and supervision. XL: visualize experimental results. GW: data record and perform the analysis with constructive discussions. All authors contributed to the article and approved the submitted version.

## Conflict of Interest

The authors declare that the research was conducted in the absence of any commercial or financial relationships that could be construed as a potential conflict of interest.

## Footnotes

^1^Grosjean R. Self-propelled high clearance vehicle: US, 8240409 B2[P], 2012-08-14.

^2^Bittner R A, Ridenour M J, Jennings R E. High clearance vehicle: US, 6454294 B1[P], 2002-09-24.

^3^Hiddema J. Agricultural application machine with variable width track. US, 8376078 B2[P], 2013-02-19.
